# Local soft tissue thickness is a superior predictor of accuracy of implant alignment compared to body mass index in total knee arthroplasty

**DOI:** 10.1186/s42836-025-00347-6

**Published:** 2025-12-02

**Authors:** Moses K. D. El Kayali, Lorenz Pichler, Clemens Gwinner, Tom Folkerts

**Affiliations:** 1 Charité – Universitätsmedizin Berlin, Center for Musculoskeletal Surgery, 10117 Berlin, Germany; 2 Department of Orthopedics and Trauma-Surgery, Medical University of Vienna, 1090 Vienna, Austria

**Keywords:** Total knee arthroplasty, Implant alignment, Local soft tissue thickness, Alignment

## Abstract

**Background:**

Precise implant positioning is critical for optimizing outcomes and implant longevity in total knee arthroplasty (TKA). Although body mass index (BMI) has been linked to postoperative complications, its association with implant malalignment remains inconclusive. As local soft tissue thickness (LSTT) may serve as a more relevant risk factor, this study aimed to evaluate the relationship between LSTT and implant alignment deviation and to compare its predictive value to BMI in conventional, manual TKA.

**Methods:**

A total of 122 consecutive patients who underwent primary TKA using the ATTUNE Knee System were retrospectively analyzed. Preoperative local soft tissue thickness was assessed at three anatomical levels: the ankle (ankle–adipose–index [AAI]), pretubercular region (pretibial subcutaneous fat [PSF]), and knee joint (knee–adipose–index [KAI]) using standardized radiographs. Postoperative implant positioning was measured by assessing coronal and sagittal alignment parameters. Group comparisons, correlation analyses, and receiver operating characteristic (ROC) curve analysis were performed to investigate associations between LSTT, BMI, and alignment deviations.

**Results:**

PSF showed a statistically significant moderate positive correlation with deviation from target proximal tibial angle (PTA) (*r* = 0.422, *P* = 0.043) and sagittal femoral angle (SFA) (*r* = 0.431, *P* = 0.041). Patients classified as outliers with alignment deviations greater than 3° had significantly higher PSF values for PTA and posterior slope angle (PSA) (*P* < 0.001). ROC analysis of pooled data identified an optimal PSF threshold of 10.9 mm to predict outliers (AUC = 0.59, sensitivity 66.7%, specificity 50.4%). No significant associations were found between AAI, KAI, or BMI and implant positioning.

**Conclusion:**

PSF is a significant risk factor for component malalignment in both coronal and sagittal planes and provides a simple, radiograph-based measure that outperforms BMI in predicting alignment accuracy in conventional TKA. A PSF threshold of approximately 11 mm may help preoperatively identify patients at risk for malalignment, in whom navigation, robotics, or patient-specific instrumentation may be considered. Further prospective studies with larger cohorts and clinical outcomes are warranted to validate these findings.

Video Abstract

**Supplementary Information:**

The online version contains supplementary material available at 10.1186/s42836-025-00347-6.

## Introduction

Total knee arthroplasty (TKA) is a highly effective treatment for end-stage osteoarthritis, providing reliable pain relief, improved function, and long-term implant durability, with annual procedure volumes expected to approach 3.5 million by 2030 [1–3].

Although TKA is associated with a high success rate, aseptic loosening and periprosthetic joint infection (PJI) continue to be the most common indications for revision surgery, affecting a substantial number of patients [4–6].

While patient-specific risk factors such as age, comorbidities, and bone quality contribute to implant failure, surgeon-related factors also play a critical role and represent important opportunities for improving implant longevity [6–9].

Among these, the accuracy of prosthetic implant alignment is a key determinant of long-term implant survival, as malalignment has been consistently associated with premature mechanical failure and poor clinical outcomes [10–17]. Various factors have been studied for their impact on alignment accuracy, including surgeon experience and volume, training status, and the use of navigation or robotic assistance [16, 18, 19].

Obesity, as reflected by an elevated body mass index (BMI), has been identified as a patient-specific risk factor associated with increased rates of postoperative complications, including impaired wound healing, infection, and implant loosening following TKA [20–22]. However, the relationship between obesity and implant positioning remains inconclusive, with existing studies reporting heterogeneous and conflicting results. [20, 21, 23–27].

A potential explanation for the challenges in achieving precise implant positioning in overweight patients is the increased local soft tissue thickness (LSTT), which may hinder adequate surgical exposure and obscure bony landmarks required for accurate placement of extramedullary alignment guides [20, 28]. Consequently, BMI is increasingly viewed as an inadequate metric for risk stratification in total joint arthroplasty (TJA), as it does not account for individual variations in fat distribution and muscular composition [29–31].

Recent studies have proposed LSTT as a potentially more relevant parameter, with retrospective analyses demonstrating associations between increased LSTT and higher rates of postoperative infection and revision surgery [29, 30, 32–34]. These findings suggest that LSTT may serve as a more precise predictor of surgical risk compared to BMI.

To date, only a limited number of studies have investigated the impact of LSTT on implant positioning in TKA [28, 35], using heterogeneous measurement methods that primarily focus on the LSTT around the knee joint.

Therefore, this study aims to evaluate whether increased LSTT hinders accurate component positioning and contributes to greater deviations from the intended alignment by analyzing multiple LSTT parameters across different anatomical regions of the lower limb. Furthermore, the study examines whether LSTT serves as a more relevant predictor of malalignment compared to BMI.

## Methods

### Patients

A total of 122 consecutive patients (68 [56%] women and 54 [44%] men) who underwent conventional, non-robotic-assisted TKA at our academic orthopaedic surgery center between October 2021 and March 2023 were included.

Inclusion criteria were defined as follows: primary TKA using the Attune Knee System (DePuy Synthes, Raynham, MA, USA) performed on either the left or right knee, availability of preoperative and postoperative radiographs, complete patient records, and documented informed consent.

**Fig. 1:**
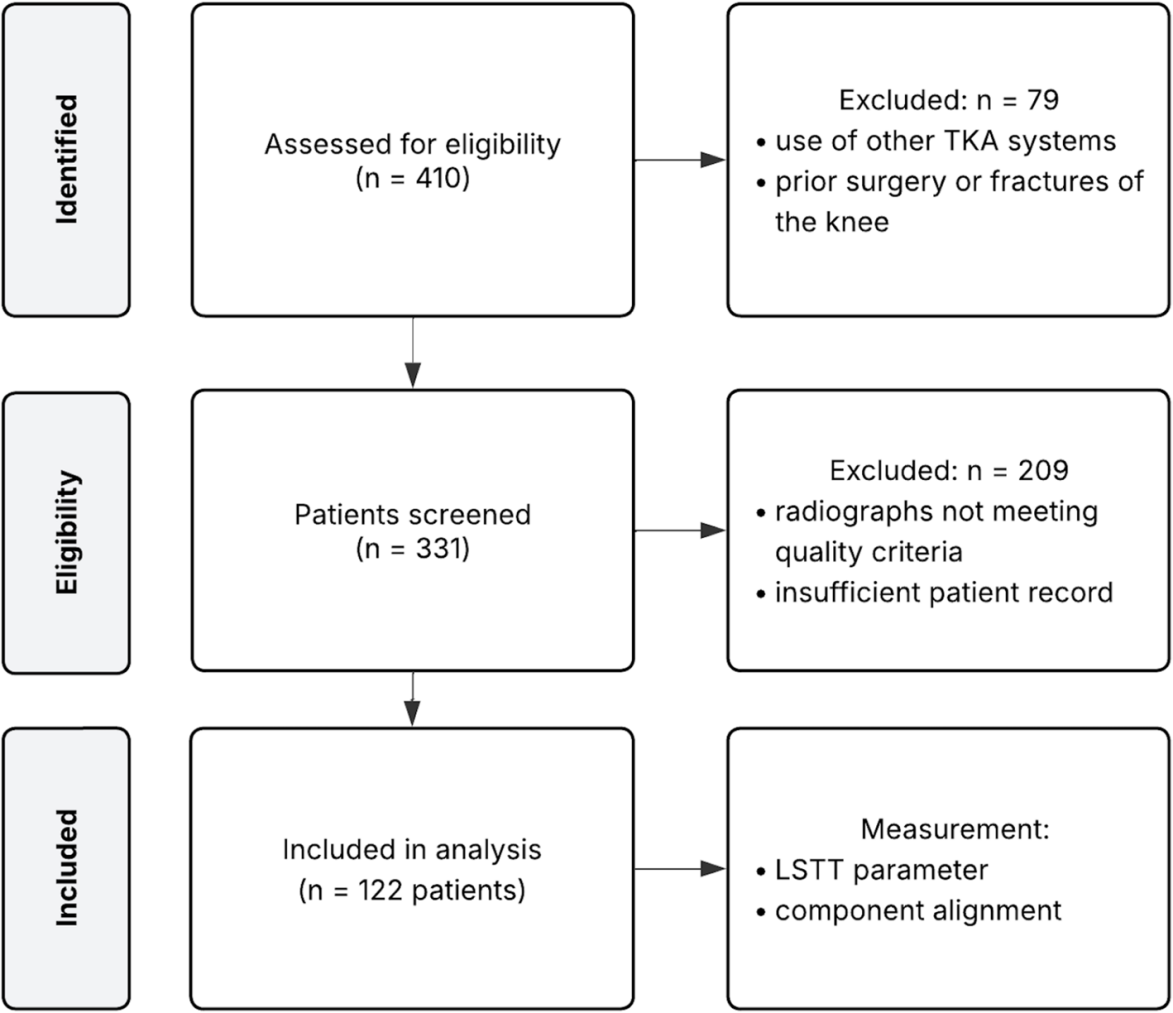
Flow diagram of patient selection and inclusion. LSTT = local soft tissue thickness; TKA = total knee arthroplasty

Exclusion criteria included radiographs that did not meet the image quality standards described in the “ Radiographs” section, insufficient patient records, history of previous ligamentous surgery or osteotomies, and the use of other TKA systems. A CONSORT-style flow diagram of patient selection is shown in Fig. 1.

Collected data included demographic variables (age, sex, BMI), surgical parameters (data of surgery, implant type, operated side), as well as preoperative and postoperative radiographs. An overview of the demographic and surgical data is provided in Table 1.

**Table 1:** Patient demographics and surgical technique

Parameter	Women (*n* = 68; 56%)	Men (*n* = 54; 44%)	Overall (*n* = 122)
***Patient demographics***			
Age (years)	72.7 (range, 56.3 to 89.3)	69.4 (range, 55.4 to 86.2)	71.3 (range, 55.4 to 89.3)
BMI (kg/m^2^)	29.3 (range, 19.3 to 42.5)	30.3 (range, 21.3 to 44.9)	29.8 (range, 19.3 to 44.9)
***Surgical technique***			
Cemented	64 (94%)	38 (70%)	102 (84%)
Cementless	4 (6%)	16 (30%)	20 (16%)

Values presented represent means if not stated otherwise

BMI = body mass index

### Surgical technique

All included cases were performed using the INTUITION (DePuy Synthes, Raynham, MA, USA) instrumentation for the ATTUNE Knee System (DePuy Synthes, Raynham, MA, USA) and in accordance with the surgical technique guidelines provided by the manufacturer. A standard parapatellar approach to the knee, a tourniquet, cruciate retaining (CR) design, and fixation by cementation or press-fit were applied in all cases. Bone resections were performed using the manufacturer’s intramedullary alignment guide for the distal femur and extramedullary guide for the proximal tibia, aiming for conventional mechanical alignment with femoral and tibial bone cuts made perpendicular to their respective mechanical axes [36]. Rotation was set using the measured resection approach. The surgical goal was to achieve a neutral mechanical axis of the femur and tibia within ± 3° in the coronal plane. In the sagittal plane, target alignment included 0° to 3° of femoral component flexion and a posterior tibial slope of 5°–7°.

### Radiographs

Lateral weight-bearing and anterior–posterior (a.-p.) long-leg weight-bearing radiographs of the operated knee were obtained both at the time of indication for TKA and postoperatively. All postoperative radiographs were acquired during the early postoperative period, within ten days post-surgery, and prior to hospital discharge.

Lateral radiographs were standardized by positioning at flexion, aligning the detector parallel to the sagittal plane, and directing the central beam at the patellofemoral joint line. For long-leg radiographs, standardization involved full knee extension, feet positioned 10 cm apart with 10° of external rotation using a positioning template with equal weight distribution between both legs.

Both preoperative and postoperative radiographs were required to meet specific inclusion criteria. For lateral views, these included a minimum visible femoral and tibial length of 15 cm, rotational alignment within 5 mm at the posterior femoral condyles, and coronal alignment with abduction or adduction deviation not exceeding 5 mm at the distal femoral condyles [37, 38]. For a.-p. views, exclusion criteria included excessive knee rotation, defined as a non-centered patella and fibular overlap exceeding one-third of its width. Only radiographs that fully captured the surrounding soft tissue with clearly defined soft tissue outlines were included for analysis.

All radiographs were calibrated using a 25.4 mm (1 inch) standard reference ball. Imaging was performed using a digital radiography system (XGEO GC85A, Samsung, Seoul, South Korea).

### LSTT measurement

LSTT was assessed preoperatively using three parameters measured at distinct anatomical regions: the ankle (ankle–adipose–index [AAI]), the pretubercular area (pretibial subcutaneous fat [PSF] [39]), and the knee joint (knee–adipose–index [KAI] [40]). These three regions were selected because they correspond to key anatomical landmarks involved in TKA: the ankle region is used for placement of the extramedullary tibial alignment clamp, the tibial tubercle region serves as a reference point for rotational alignment and instrument positioning, and the knee joint region, as it is the direct site of prosthetic component placement.

**Fig. 2:**
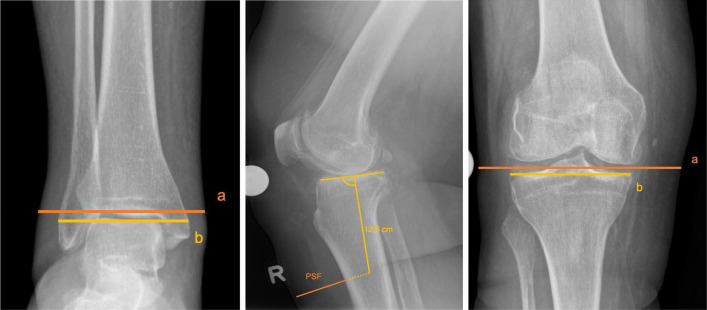
Measurement of the Local Soft Tissue Thickness (LSTT). Representative images demonstrating the measurement of LSTT parameters on preoperative radiographs of a right knee are presented from left to right. The ankle-adipose-index (AAI) was defined as the ratio of the total thickness of the ankle at the level of the malleoli (**a**) to the bony width of the ankle (**b**) (left image). The pretibial subcutaneous fat (PSF) is measured as the distance from the anterior cortex of the tibia to the overlying skin, using a perpendicular line drawn 12.5 cm distal to the tibial plateau (central image). The knee-adipose-Index (KAI) was calculated as the ratio of the total soft tissue thickness (**a**) to the width of the tibial plateau (**b**) (right image)

The AAI was defined as the ratio of the total thickness of the ankle at the level of the malleoli to the bony width of the ankle, as measured on a.-p. radiographs. The PSF was measured on lateral radiographs as the distance from the anterior cortex of the tibia to the overlying skin, using a perpendicular line drawn 12.5 cm distal to the tibial plateau [39]. The KAI was calculated as the ratio of the total soft tissue thickness to the width of the tibial plateau on a.-p. radiographs, specifically defined as the total thickness of the leg at the level of the tibial plateau, divided by the bony width of the tibial plateau [40]. Osteophytes, defined as bony projections extending beyond the normal cortical margins at the joint surface, were excluded from all measurements. LSTT measurements are illustrated in Fig. 2.

### Measurement of component alignment

**Fig. 3:**
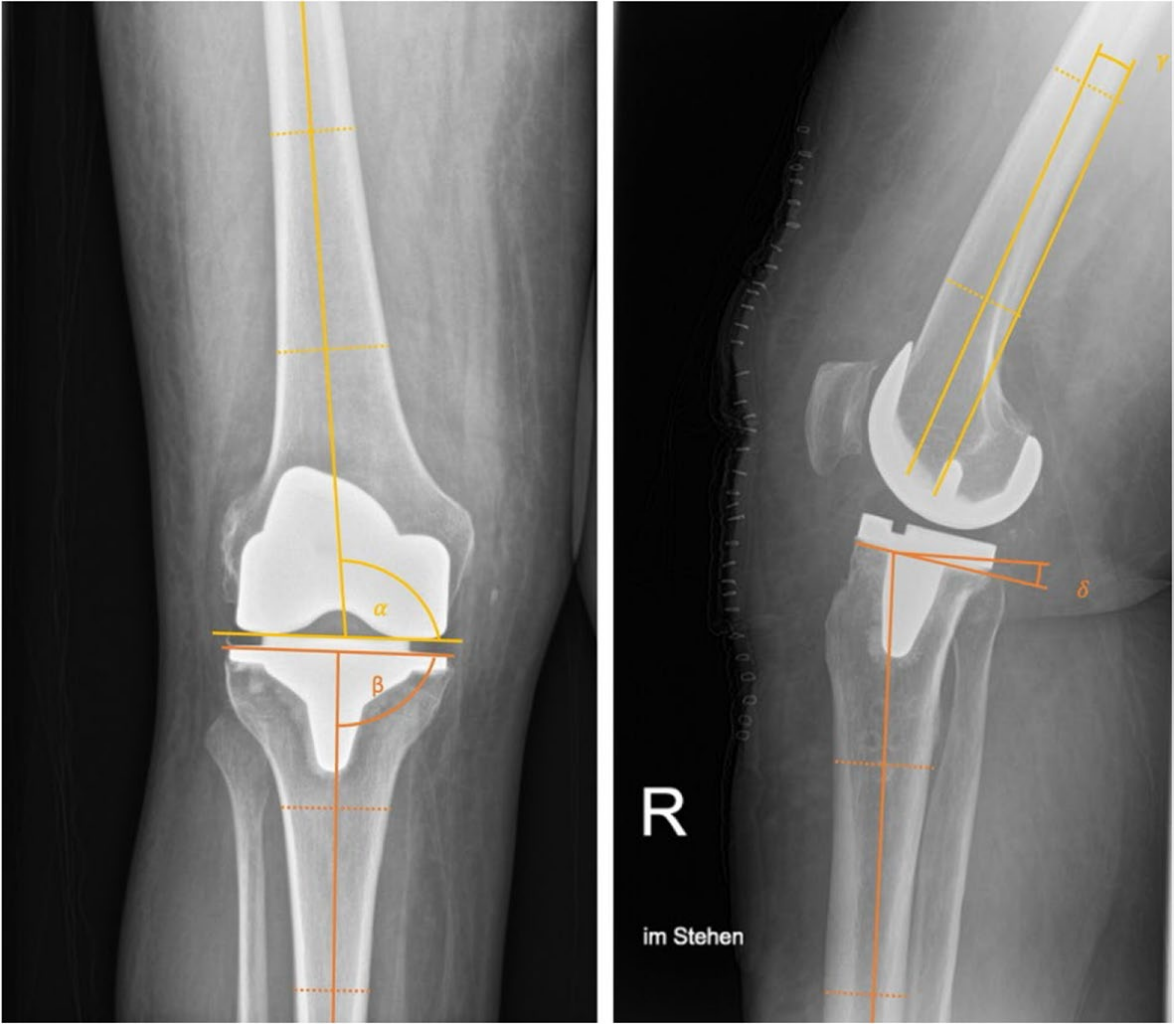
Measurement of component alignment. Representative images demonstrating the measurement of component alignment parameters on postoperative radiographs of a right knee. The Distal Femoral Angle (DFA, α) was defined as the medial angle between the femoral anatomical axis and the distal surface of the femoral component (left image). The Proximal Tibial Angle (PTA, β) was measured as the medial angle between the tibial anatomical axis and a line parallel to the tibial baseplate (left image). The Sagittal Femoral Angle (SFA, γ) was defined as the angle between the most distal femoral fixation surface in relation to the femoral shaft axis (right image). The Posterior Slope Angle (PSA, δ) was defined as the angle formed between the tibial anatomical axis and the tibial baseplate (right image)

Coronal and sagittal alignment of the femoral and tibial components was measured according to previously described methods [18, 41, 42] on postoperative radiographs (Fig. 3).

For coronal alignment, the Distal Femoral Angle (DFA, α) was defined as the medial angle between the femoral anatomical axis and the distal surface of the femoral component, while the Proximal Tibial Angle (PTA, β) was measured as the medial angle between the tibial anatomical axis and a line parallel to the tibial baseplate. For sagittal alignment, the Sagittal Femoral Angle (SFA, γ) was defined as the angle between the most distal femoral fixation surface in relation to the femoral shaft axis. The Posterior Slope Angle (PSA, δ) was evaluated on lateral radiographs as the angle formed between the tibial anatomical axis and the tibial baseplate.

Two independent observers (T.F., M.E.), blinded to patient clinical data, performed all measurements of alignment parameters and LSTT. Each observer repeated the measurements twice, with a minimum interval of 14 days between assessments. Intra-class correlation coefficients (ICCs) for both inter- and intra-observer reliability demonstrated excellent agreement (>0.8) for all assessed parameters, including component alignment and LSTT measurements [43].

All measurements were performed using a PACS workstation (Centricity RIS-I 4.2 Plus, GE Healthcare, Chicago, IL, USA).

### Data analyses

Descriptive parameters were calculated, including mean, standard deviation, median, and range, as appropriate. The Shapiro–Wilk test was used to assess the normality of quantitative data. For normally distributed data, comparisons were performed using a two-sided t-test. Non-normally distributed data were analyzed using the Mann–Whitney U test for independent samples and the Wilcoxon signed-rank test for dependent samples. Correlations were assessed using Spearman’s rank correlation coefficient for non-normally distributed data and Pearson’s correlation coefficient for normally distributed data. For all correlation analyses, both the correlation coefficient (*r*) and the corresponding p-value were reported. A *P*-value of less than 0.05 was considered statistically significant. Group comparisons between in-target and outlier cases were pre-specified. Because these analyses included multiple, simultaneous tests across BMI, AAI, PSF, and KAI in relation to four alignment angles, Bonferroni correction was applied, and adjusted *P*-values are reported. In contrast, the correlation analyses between LSTT parameters, BMI, and alignment angles were exploratory; *P*-values are therefore reported unadjusted and should be interpreted as hypothesis-generating. A correlation coefficient (*r*) of 0–0.19 was regarded as very weak, 0.2–0.39 as weak, 0.40–0.59 as moderate, 0.6–0.79 as strong, and 0.8–1 as very strong correlation [44].

Descriptive parameters were calculated for all LSTT parameters, BMI, and mean implant positioning values. Spearman correlation coefficients were calculated to assess associations between the individual LSTT parameters, as well as between each LSTT parameter and BMI. Additional Spearman correlations were performed to evaluate the relationship between LSTT, parameters, BMI, and the deviation from the target alignment as defined under ‘surgical technique’. For alignment parameters that showed a significant correlation with LSTT parameters in the univariate analysis, multivariable linear regression models were constructed to evaluate whether the LSTT parameter remained an independent predictor after adjusting for potential confounders. Covariates included age, sex, BMI, and surgeon. Model assumptions were checked and met. Cases with a deviation of 3° or more from the intended alignment were defined as outliers [41, 45–47]. The mean values of each LSTT parameter and BMI were compared between the outlier group and the in-target group using the Mann–Whitney U test. For alignment parameters where the group comparison (in-target vs. outlier) showed a statistically significant difference in LSTT, we performed receiver operating characteristic (ROC) curve analyses to derive clinically applicable cut-off values for the LSTT parameter. Outlier status (≥3° deviation) served as the binary outcome. The area under the curve (AUC), sensitivity, specificity, and optimal cut-off values were determined using Youden’s J statistic [48].

All calculations were performed using Microsoft Excel for Mac (Version 16.95.4, Microsoft, Redmond, WA, USA) and SPSS (IBM Corp. Released 2023. IBM SPSS Statistics for Windows, Version 29.0.2.0, Armonk, NY: IBM Corp).

### Ethical aspects

The study protocol was approved by the institutional ethics committee of Charité – Universitätsmedizin Berlin (EA2/016/21) and conducted in accordance with the principles of the Declaration of Helsinki. Written informed consent was obtained from all participants.

## Results

Of the 410 patients who underwent TKA at our center during the study period, 122 (30%) met the inclusion criteria and were included for analysis.

Mean values for LSTT parameters are presented in Table 2. Spearman correlation analysis revealed statistically significant moderate to strong positive correlations between the individual LSTT parameters, as well as between each LSTT parameter and BMI (AAI and KAI: *r* = 0.461, *P* < 0.001; AAI and PSF: *r* = 0.491, *P* < 0.001; KAI and PSF: *r* = 0.702, *P* < 0.001; AAI and BMI: *r* = 0.452, *P* < 0.001; PSF and BMI: *r* = 0.559, *P* < 0.001; KAI and BMI: *r* = 0.585, *P* < 0.001).

**Table 2:** Preoperative local soft tissue thickness (LSTT)

Parameter (mm)	Mean (mm) ± SD
AAI	1.19 ± 0.129
PSF	14.2 ± 8.54
KAI	1.70 ± 0.256

Values presented represent means if not stated otherwise

AAI = ankle-adipose-index; PSF = pretibial subcutaneous fat; KAI = knee-adipose-index

Mean postoperative alignment parameters, including DFA, PTA, SFA, and PSA, are summarized in Table 3. The proportion of cases classified as outliers for each alignment parameter is presented in Table 4.

**Table 3:** Postoperative alignment parameters

Parameter	Mean ± SD
DFA, α (°)	91.84 ± 2.27
PTA, β (°)	89.27 ± 2.23
SFA, γ (°)	2.93 ± 2.77
PSA, δ (°)	6.29 ± 2.47

Values presented represent means if not stated otherwise

DFA = distal femoral angle; PTA = proximal tibial angle; SFA = sagittal femoral angle; PSA = posterior slope angle

**Table 4:** Distribution of outliers according to postoperative alignment parameters

Parameter	Outliers	
	***n***	**%**
DFA, α	22	18.03
PTA, β	25	20.49
SFA, γ	18	14.75
PSA, δ	23	18.85

Values presented represent means if not stated otherwise

DFA = distal femoral angle; PTA = proximal tibial angle; SFA = sagittal femoral angle; PSA = posterior slope angle

Spearman correlation analysis demonstrated a statistically significant moderate positive correlation between PSF and the deviation from the target PTA (*r* = 0.422, 95% CI 0.26–0.56, *P* = 0.043), as well as a moderate statistically significant positive correlation between PSF and deviation from the target SFA (*r* = 0.431, CI 0.27–0.57, *P* = 0.041). In multivariable regression analyses adjusting for age, sex, BMI, and surgeon, PSF remained significantly associated with deviation from PTA (β = 0.21, 95% CI 0.08–0.34, *P* = 0.002) and SFA (β = 0.19, 95% CI 0.05–0.33, *P* = 0.008). No significant correlations were observed between AAI, KAI, or BMI with any alignment parameters. Detailed results are presented in Table 5.

**Table 5:** Spearman correlation between local soft tissue thickness (LSTT) parameters and deviation from implant positioning target

Parameter	DFA, α		PTA, β		SFA, γ		PSA, δ	
	**r**	***P***	**r**	***P***	**r**	***P***	**r**	***P***
**BMI**	−0.002	0.988	0.118	0.287	−0.017	0.877	0.064	0.567
**AAI**	0.108	0.332	0.002	0.989	−0.135	0.224	−0.031	0.782
**PSF**	−0.02	0.855	0.422	0.043*	0.431	0.041*	0.113	0.309
**KAI**	−0.053	0.634	0.138	0.212	0.033	0.768	0.116	0.297

^*^statistical significance. *P*-values are unadjusted and exploratory

BMI = body mass index; AAI = ankle-adipose-index; PSF = pretibial subcutaneous fat; KAI = knee-adipose-index

Comparison of mean LSTT and BMI values between the outlier group and the in-target group revealed a statistically significant difference in PSF for both PTA (*P* < 0.001, Cohen’s d = 0.37) and PSA (*P* < 0.001, Cohen’s d = 0.78). No significant differences were observed for other LSTT parameters or for BMI. These findings are summarized in Table 6. ROC analysis of PSF for predicting alignment outliers identified the following thresholds: for PTA outliers, 10.9 mm (AUC = 0.59, sensitivity 67.9%, specificity 54.5%); for PSA outliers, 17.0 mm (AUC = 0.66, sensitivity 60.0%, specificity 76.9%). When pooling both parameters, a unified PSF threshold of 10.9 mm provided the best discrimination (AUC = 0.59, sensitivity 66.7%, specificity 50.4%).

**Table 6:** Comparison of preoperative LSTT, BMI, and postoperative implant positioning between the outlier group and the in-target group

Parameter	BMI (Outliers)	BMI (in-target)	*P*-Value	AAI (Outliers)	AAI (in-target)	*P*-Value	Mean PSF (Outliers)	Mean PSF (in-target)	*P*-Value	Mean KAI (Outliers)	Mean KAI (in-target)	*P*-Value
DFA, α (°)	29.76	29.80	>0.05	1.21	1.18	>0.05	16.15	14.25	>0.05	1.69	1.70	>0.05
PTA, β (°)	30.14	29.60	>0.05	1.18	1.19	>0.05	16.09	13.25	< 0.001*	1.71	1.69	>0.05
SFA, γ (°)	31.41	29.58	>0.05	1.19	1.19	>0.05	15.33	14.19	>0.05	1.81	1.68	>0.05
PSA, δ (°)	27.96	29.77	>0.05	1.15	1.19	>0.05	20.23	13.89	< 0.001*	1.78	1.68	>0.05

Values presented represent means if not stated otherwise. Bonferroni’s correction applied. *statistical significance

BMI = body mass index; LSTT = local soft tissue thickness; AAI = ankle-adipose-index; PSF = pretibial subcutaneous fat; KAI = knee-adipose-index

## Discussion

This study aimed to investigate whether increased LSTT is associated with deviations from target implant alignment in TKA, and whether LSTT serves as a more reliable predictor of malalignment compared to BMI. Two key findings emerged in support of these hypotheses. First, PSF was significantly correlated with deviation from the intended PTA and SFA, and was notably higher in outlier cases for both PTA and PSA, suggesting an influence on component positioning in both planes. Second, neither the other LSTT parameters nor BMI showed significant associations with alignment deviation, indicating that PSF may be the most relevant soft tissue-related risk factor for implant malpositioning, while BMI alone lacks predictive value in this context.

The precise positioning of TKA components is crucial for optimizing clinical outcomes, as it enhances joint stability, reduces the risk of implant loosening, lowers revision rates, and prolongs the overall longevity of the prosthesis [14, 49–52]. This correlation has been supported by Choong et al., who demonstrated that achieving a mechanical axis within 3° of neutral in TKA is associated with superior functional outcomes and improved quality of life [16]. In a randomized prospective trial, patients with well-aligned knees demonstrated significantly superior functional outcomes, as assessed using International Knee Society (IKS) and SF-12 scores, across all follow-up intervals from 6 weeks to 12 months [51]. Supporting these findings, Longstaff et al. identified that patients with lower cumulative alignment deviations had superior postoperative functional recovery, as indicated by improved mobility, reduced pain, greater knee stability, and a shorter rehabilitation period. [52] Furthermore, a study by Fang et al. revealed that TKA survival rates were highest in patients with neutral alignment, as indicated by the lowest revision rate (0.5%), whereas varus (1.8%) and valgus (1.5%) malalignment were associated with significantly increased failure rates [49]. The definition of outliers as ≥ 3° deviation was based on prior studies linking this threshold to functional outcomes and implant survival [41, 45–47]. While more modern unrestricted kinematic alignment strategies advocate for restoring each patient’s constitutional limb alignment, even when it lies outside the conventional ± 3° “safe zone”, current evidence remains inconclusive [53]. Although some studies have demonstrated comparable short- to mid-term outcomes, the overall body of evidence does not indicate a clear clinical advantage of these unrestricted alignment strategies over mechanically aligned TKA [54–56]. Mechanically aligned TKA within ±3° of neutral alignment, therefore, remains the current gold standard for achieving reliable functional outcomes and implant longevity [56].

The impact of obesity, as indicated by an elevated BMI, on implant positioning in TKA remains a subject of conflicting findings [20, 21, 23, 24]. Several studies provide evidence supporting a significant impact of BMI on postoperative implant positioning. For instance, Estes et al. [20] reported that an increase in BMI was associated with a higher risk of postoperative malalignment. Similarly, Krushell and Fingeroth [21] found a significant difference in femoral-tibial alignment when comparing morbidly obese (BMI > 40 kg/m^2^) and matched non-obese patients (BMI < 30 kg/m^2^), with a shift toward varus malalignment in the obese group. In contrast, studies conducted by Compton et al. [23] and Shetty et al. [24] failed to identify any significant association between BMI and implant positioning in TKA. Consistent with the latter findings, the present study also demonstrated no correlation between BMI and alignment deviation in TKA.

Given the well-known limitations of BMI, including its inability to reflect body fat distribution or distinguish between fat mass and fat-free mass, numerous studies have investigated LSTT as a potentially more reliable predictor of complications in TKA [31, 57]. However, analogous to the heterogeneous findings reported for BMI, the literature evaluating LSTT as a predictive marker of postoperative complications following TKA demonstrates substantial variability. Wagner et al. [30] demonstrated that a higher prepatellar fat thickness ratio (PFTR) was associated with an increased risk of postoperative infections. Furthermore, the PFTR has been identified as a more effective predictor of infections than BMI [29, 30]. Similarly, different studies observed that increased soft tissue thickness was associated with a higher risk of periprosthetic joint infection (PJI), independent of BMI [29, 33]. Consistent with these findings, Yu et al. [34] investigated the periarticular soft tissue index (PASTI) and identified a significantly higher rate of wound healing complications in patients with an elevated PASTI value, whereas BMI had no significant impact. Correspondingly, Watts et al. [32] discovered that a prepatellar thickness of ≥15 mm and a pretubercular thickness of ≥25 mm were significantly associated with an elevated risk of early reoperation. In contrast, Gupta et al. [58], Secrist et al. [35], and Shearer et al. [40] found no significant association between increased LSTT and an elevated risk of postoperative complications such as infections.

Although a substantial body of literature has examined infectious complications and reoperation rates, the influence of LSTT on implant positioning in TKA remains largely underexamined. Secrist et al. [35] examined the relationship between Lower Extremity Girth (LEG) and implant positioning in a cohort of TKA patients. The LEG ratio was determined from lateral knee radiographs by dividing the width of the soft tissue envelope by the bone width 2 cm above the posterior femoral condyles. No significant correlation was observed between the LEG ratio and malalignment. Another study investigating the suprapatellar fat index (SPFI) as a surrogate of LSTT around the knee joint also found no association with postoperative tibial component alignment [59].

Similar to the LEG ratio and the SPFI, KAI, which was measured in our study, did not show a significant association with alignment deviation in our cohort, supporting the finding that the amount of local soft tissue at the level of the knee joint does not appear to significantly impact implant positioning in TKA.

In contrast to these previous studies that focused exclusively on the LSTT around the knee joint, our study expanded the analysis to include two additional anatomical levels: the pretubercular region (PSF) and the ankle region (AAI). These sites were selected because they correspond to commonly used intraoperative reference points and for the placement of extramedullary alignment guides during TKA. The moderate to strong correlations between the LSTT parameters and BMI found in our study suggest that higher BMI is generally associated with increased LSTT across multiple anatomical regions. The moderate to strong intercorrelation among LSTT parameters further supports the consistency of soft tissue distribution, indicating a systemic pattern of adiposity in these patients. However, the finding that these correlations did not reach very strong levels (*r* > 0.8) indicates that the parameters are not fully collinear and may reflect distinct anatomical aspects of soft tissue distribution.

Interestingly, PSF emerged as a significant risk factor for implant malpositioning in both the coronal and sagittal planes, affecting alignment of both the femoral and tibial components, as statistically significant correlations were observed between PSA and deviation from the target values for PTA and SFA. Importantly, this association remained significant in multivariable regression models adjusting for age, sex, BMI, and surgeon, confirming PSF as an independent predictor of both coronal and sagittal malalignment. Supporting this finding, cases classified as outliers with 3° or more of deviation from the target alignment demonstrated significantly higher PSF values for both PTA and the PSA. According to the ATTUNE Surgical Technique Manual, tibial resection is performed with the knee in 90° of flexion, with the ankle clamp positioned around the malleoli and varus/valgus-alignment set by referencing the proximal central marking on the tibial cutting block to the medial one-third of the tibial tubercle. In patients with increased PSF, this referencing step may be comprised, as the prominent soft tissue envelope may obscure or displace the visual and tactile landmark required for precise guide positioning. This is supported by research showing that increased thigh size is associated with longer tourniquet times during TKA, suggesting a higher degree of intraoperative complexity in patients with greater LSTT [60]. While this explanation may account for the observed malalignment in tibial component positioning, the correlation between PSF and SFA, despite the use of an intramedullary femoral guide, may be attributed to the impaired surgical exposure caused by increased LSTT. Excessive LSTT may limit visualization of key femoral landmarks such as the transepicondylar axis, which several studies have shown to be challenging for surgeons to identify accurately and reproducibly [61–64]. This could contribute to minor deviations during femoral resection, even when intramedullary referencing is employed. Thus, our findings suggest that excessive LSTT may influence not only extramedullary tibial alignment but also the accuracy of femoral component positioning, likely through indirect mechanisms related to surgical access and field visualization. Conversely, the LSTT at the ankle region, represented by the AAI, showed no associations with alignment deviation in any plane, indicating that not all LSTT parameters contribute to the accuracy of implant positioning.

Accurate assessment of risk for complications and readmission is essential for optimizing outcomes after TJA [65]. Based on our findings, preoperative counseling for TKA should address not only increased BMI but also increased LSTT as a potential risk factor for technical difficulty and implant malalignment. Preoperative radiographic assessment of LSTT may serve as a useful screening tool to identify patients at risk. Supporting this, ROC analysis in our cohort suggested a PSF threshold of approximately 11 mm as a potential indicator of increased risk for malalignment (AUC = 0.59, sensitivity 66.7%, specificity 50.4%). In such cases, the use of techniques that have been shown to increase the accuracy of implant alignment, such as navigation, robotic assistance, or patient-specific instrumentation, may be considered [24, 66–71]. However, given the limited discriminatory ability of this threshold, PSF should be interpreted as a risk marker rather than a strict cut-off for clinical decision-making.

This study has several limitations in addition to those inherent to its retrospective design. The exclusive use of a single TKA system may limit generalizability, although it also eliminates variability related to different instrumentation systems. The absence of PROMs, revision rates, or other long-term outcome data prevents us from directly linking the observed radiographic findings to clinical benefit or failure. As such, the clinical interpretation of our results must remain cautious. Another limitation is that LSTT measurements were based solely on two-dimensional radiographs, which have lower soft tissue resolution compared to advanced imaging modalities such as computed tomography, magnetic resonance imaging, or ultrasound. Although only high-quality radiographs with clearly visible soft tissue margins were included, some degree of measurement imprecision remains inherent to plain radiography. The strict radiograph quality criteria applied, including exclusion of cases with rotation, abduction/adduction malalignment, or non-centered patella, led to the exclusion of a large proportion of patients and may introduce selection bias despite ensuring high measurement accuracy. Moreover, while magnetic resonance imaging or ultrasound could provide more precise soft tissue quantification and differentiate between skin and subcutaneous fat layers, these modalities are not performed routinely prior to TKA in our setting. Thus, validation of PSF against such advanced imaging was not feasible in this study, but future work should aim to address this. Furthermore, the relatively small sample size, particularly in the outlier groups, may reduce the statistical power of subgroup analyses such as t-tests. However, the results of group comparison were consistent with the correlation analyses, supporting the internal validity of the findings. An additional limitation is that not all planes of knee alignment were assessed in this study, as rotational alignment in the axial plane was not included in the analysis. Also, surgeons were naturally aware of patients’ BMI and general body habitus, which could have influenced surgical exposure and technique, thereby introducing some degree of performance bias. However, the radiographic PSF values were not known intraoperatively, and therefore, this specific parameter could not have directly biased surgical alignment. Finally, comparison with other studies on LSTT remains challenging due to the absence of a universally accepted reference standard and the use of heterogeneous measurement methods across the literature.

Further studies should aim to validate these findings in larger, prospective patient cohorts and incorporate clinical outcome data, including PROMS and implant survival, to fully assess the clinical impact of LSTT on outcomes after TKA.

## Conclusion

This study identified the PSF as a significant risk factor for component malalignment in both the coronal and sagittal planes, confirming that LSTT is a more accurate predictor of postoperative implant positioning than BMI in conventional, non-robotic-assisted TKA. ROC analysis suggested a PSF threshold of approximately 11 mm as a potential indicator of increased risk for malalignment. Radiographic assessment of PSF on standard lateral radiographs may therefore serve as a simple screening tool to identify patients at risk, in whom the use of alignment-optimizing techniques such as computer navigation, robotic assistance, or patient-specific instrumentation may be considered. Further research involving larger patient cohorts and incorporating clinical outcome measures is needed to validate these findings and to fully assess the clinical relevance of PSF in TKA outcomes.

## Data Availability

The datasets generated and analyzed during the current study are available from the corresponding author upon reasonable request.

## Abbreviations

AAI: Ankle Adipose Index
AUC: Area under the curve
A.-P.: Anterior–Posterior
BMI: Body Mass Index
CR: Cruciate-retaining
DFA: Distal Femoral Angle
ICC: Intraclass Correlation Coefficient
IKS: International Knee Society
KAI: Knee Adipose Index
LEG: Lower Extremity Girth
LSTT: Local Soft Tissue Thickness
MA: Mechanical Alignment
PASTI: Periarticular Soft Tissue Index
PFTR: Prepatellar Fat Thickness Ratio
PJI: Periprosthetic Joint Infection
PROMs: Patient-Reported Outcome Measures
PSA: Posterior Slope Angle
PSF: Pretibial Subcutaneous Fat
PTA: Proximal Tibial Angle
ROC: Receiver operating characteristic
SFA: Sagittal Femoral Angle
SPFI: Suprapatellar Fat Index
TJA: Total Joint Arthroplasty
TKA: Total Knee Arthroplasty
